# Challenges and management of venomous bites and scorpion stings in Lebanon: a qualitative study

**DOI:** 10.3389/fpubh.2025.1585250

**Published:** 2025-06-02

**Authors:** Kamil Kadi, Tharwat El-Zahran, Mario Badr, Mohammad Mdaihly, Nesrine Ahmad, Atika Berri, Michelle E. E. Bauer, Samar Al-Hajj

**Affiliations:** ^1^Faculty of Medicine, American University of Beirut Medical Center, Beirut, Lebanon; ^2^Department of Emergency, American University of Beirut Medical Center, Beirut, Lebanon; ^3^MENA Program for Advanced Injury Research, Faculty of Health Sciences, American University of Beirut, Beirut, Lebanon; ^4^Ministry of Public Health, Beirut, Lebanon; ^5^British Columbia Injury and Research Prevention Unit, Faculty of Medicine, University of British Columbia, BC, Canada; ^6^Faculty of Health Sciences, Department of Epidemiology and Population Health, American University of Beirut, Beirut, Lebanon

**Keywords:** venomous bites, Lebanon, health inequity, health management, sociocultural theory, Eastern Mediterranean region, animal bites, toxin exposure

## Abstract

**Background:**

Snakebites and scorpion stings are significant public health issues globally, particularly in the Middle East. This qualitative study investigates the management of these incidents in Lebanon by exploring the perceptions of healthcare providers and public health experts.

**Methods:**

Thematic analysis, guided by sociocultural theory, examined qualitative data from 17 interviews with healthcare providers, including emergency physicians, paramedics, pharmaceutical providers, and ministry workers. Transcripts were coded to identify recurring themes related to the management of snakebites and scorpion stings, focusing on availability, accessibility, inequity, healthcare access disparities, and cultural influences on treatment-seeking behavior.

**Results:**

The analysis revealed significant disparities in antivenom availability and accessibility, particularly in rural areas and among low socioeconomic groups. Healthcare providers often resorted to illicit channels to secure antivenom due to stock shortages, while victims sometimes relied on traditional treatment methods. The lack of standardized treatment protocols and inadequate clinician training resulted in inconsistent antivenom usage and unsafe practices. The study also highlighted insufficient documentation and reporting mechanisms and inadequate networking among stakeholders, alongside a notable knowledge gap among victims.

**Conclusion:**

This study emphasizes the urgent need for targeted interventions to address systemic challenges in managing snakebites and scorpion stings in Lebanon. Collaborative efforts are essential to enhance awareness, improve antivenom access, standardize treatment protocols, and promote effective management practices.

## 1 Introduction

Intoxication from wildlife venom is a significant public health concern, particularly where venomous species predominantly live and intersect with human activities (1). According to the World Health Organization (WHO), approximately 5.4 million people worldwide are bitten by snakes annually, resulting in 1.8 to 2.7 million cases of envenomation. Each year, an estimated 81,410 to 137,880 deaths are attributed to snake bites, along with three times as many amputations and other permanent disabilities (2). Snakebites alone account for an estimated 420,000 cases of venom-induced chemical intoxication globally, resulting in around 20,000 deaths (3, 4). Scorpion envenomation further poses a similar threat, with underreporting and regional variations, complicating the accurate assessment of the burden worldwide. This issue is particularly alarming in regions such as North Africa, the Middle East, South Asia, and Arid-Temperate regions of the Americas (5), which present the ideal habitat for snakes and scorpions with the dry weather, warm temperature, and prey availability.

The Middle East’s rich biodiversity, including various venomous snakes and scorpions, contributes to the pressing concern of snakebites and scorpion stings (4, 5). In this region, species such as Vipera palestinae, Vipera lebetina, and *Vipera bornmuelleri* are among the most reported causes of snake envenomation (3, 4, 6). Likewise, scorpion species such *as Leiurus, Androctonus, and Hottentotta judaicus* are prevalent (7). Snakebites and scorpion stings’ severity vary, from mild to life-threatening, depending on several factors including venom components, type, quantity, and individual sensitivity, necessitating appropriate medical treatment in severe cases (8). However, the accurate assessment of morbidity and mortality rates for intoxication from wildlife venom remains unknown due to challenges related to underreporting, lack of awareness, and insufficient preventive measures. Lebanon, with its geographical exposure to snake species and associated risks, stands as a unique case study (3, 4). A recent report from the Lebanese Ministry of Public Health (MoPH) documented four cases of snake envenomation between May and June of 2022 (9).

While wildlife envenomation poses immediate threats to the affected populations and further contributes to the burden of healthcare systems in these endemic regions, limited studies have examined the management of these cases in the Middle Eastern region (10, 11). In the MENA region, limited efforts have been made to develop standardized protocols for snakebite management. Existing guidelines, primarily from Oman and Saudi Arabia, are outdated, inconsistent, and largely derived from individual case reports rather than robust clinical evidence (12, 13). These protocols have remained unchanged in recent years, and national strategies have yet to be updated and published to address evolving needs. In Lebanon, only the Ministry of Public Health has issued treatment and management guidelines for snakebites (14); however, these continue to lack sufficient details with limited assessment for their implementation, effectiveness, or impact on patient outcomes.

Climate change further complicates the situation, potentially increasing the incidence of envenomation by altering habitats to favor venomous species. Scorpion stings and snake bites are fundamentally influenced by climate, environment, and human activity. As ectotherms, snakes and scorpions proliferate more active in warmer conditions (15, 16). Climate change, therefore, is expected to intensify human–snake interactions by shifting snake distributions, increasing their abundance, and altering activity patterns in response to rising temperatures and extreme weather (15–17). These environmental changes, combined with human factors— such as altered farming practices, displacement, and man-made infrastructure expansion and encroachment into natural habitats, will heighten the risk of encounters (15–17). There is limited evidence on how climate change affects snakebite burden, reflecting major gaps in understanding venomous snake distribution and their impact on humans (15, 17). A review of the available evidence shows that climate change may shift snakebite risk northward in North America and southward in South America, consistent with ecological studies showing similar changes in snake distribution, including findings from China (17). This suggests that regions and communities not currently affected by snakebite as a public health issue may experience an increase in cases in the future due to climate change. The WHO utilizes geographic information systems (GIS) and advanced modeling techniques to map snake distributions and forecast future changes in snake-human interactions due to climate change (18). Therefore, understanding the multifaceted aspects of snakebites and scorpion stings, including perspectives from healthcare providers and victims’ experiences, is crucial for devising effective prevention and treatment strategies.

This qualitative study aims to explore the clinical and medical aspects of envenomation cases in Lebanon by exploring the perceptions, experiences, and challenges encountered by healthcare providers, paramedics, and public health experts in managing snakebites and scorpion stings. Additionally, the study aims to identify potential shortcomings within the Lebanese healthcare system infrastructure, training, public awareness, emergency response, treatment availability, and the system’s capacity to adapt to changing climate conditions. The findings will offer recommendations for addressing envenomation cases and propose strategies for prevention, management, and treatment of wildlife envenomation, particularly snakebites and scorpion stings in low- and- middle-income countries. While offering valuable insights, the study is limited by the scarcity of envenomation cases, the inability to access key informants such as high-level policymakers, and the reliance on regional data due to the lack of national records. While qualitative, this study offers insightful findings and provides a strong foundation to guide future research and policy efforts.

## 2 Materials and methods

### 2.1 Study design

A qualitative study was conducted to explore health sector professionals’ perspectives on the management of resources for venomous bites and scorpion stings across Lebanon. Open-ended interviews were conducted with stakeholders in public-facing health professional roles, such as emergency physicians, who worked at the time of the interview at local hospitals or within governmental and non-governmental organizations (NGOs; Supplementary material). Participation in this research was voluntary, allowing stakeholders to respond without constraints. Before administering the interviews, explicit consent was obtained from participants for audio recording or note-taking and the utilization of responses in the transcription of the study report.

### 2.2 Theoretical framework

The study was guided by our use of sociocultural theory, which emphasizes the impact of social, cultural, and economic factors on health behaviors and outcomes (19). Previous research addressing socio-cultural factors relating to snakebites and scorpion stings has been prominent in addressing inequities in experiences and in highlighting areas of need in symptom management and medical care affordance (20, 21). Sociocultural theory was used to shape the understanding of the inequities in healthcare access in lower socio-economic status geographical catchment areas that were identified through conversations with participants and to understand the cultural practices, such as prevention beliefs, affecting the management of bites and stings in Lebanon and access to medical resources and symptom management. Socio-cultural theory thus provided a lens through which we could examine the disparities in healthcare resources afforded to the public, such as hospital and medical professional access and antivenom affordance, and the public education available for Lebanese families on snakebite and scorpion stings. The sociocultural theory informed the discussions highlighting the systemic issues that contribute to inequities in the prevention of and intervention for snakebite and scorpion sting experiences.

### 2.3 Participants

National stakeholders were selected as interview participants. A purposeful sampling approach was employed (22), through which participants’ professional backgrounds and expertise in national readiness for venomous animal bites were considered. Participants were chosen based on their pivotal roles as decision-makers in the administration and/or management of antivenom and bite management. Overall, the final sample included 17 stakeholders to achieve thematic saturation and to offer rich and diverse perspectives relevant to addressing the study aims. Participants were purposefully selected from the most affected regions in Lebanon (North and South) and included diverse specialties and leadership roles. The selected stakeholders encompassed diverse entities, including governmental and non-governmental decision-makers, the Prevention and Epidemiological Surveillance Unit at the MoPH, representatives from the Ministry of Environment, as well as professionals from tertiary and rural emergency departments (ED) at national hospitals. These included departmental heads and physicians from various fields—ED and internal medicine—from both rural and urban hospitals, a toxicologist, public health representatives, pharmacists, and Red Cross paramedics. Most of these individuals (i.e., 14 out of 17) were senior professionals with 15 to 40 years of experience in the field. Few were junior officials with less than 10 years in the field (i.e., 3 out of 17). Given the country’s small size and limited snakebite burden, this allowed for broad representation. Data saturation was reached as no new themes emerged in later interviews.

### 2.4 Data collection

Open-structured interviews were conducted in person or via video conference, by CITI-certified fourth-year medical students enrolled at the American University of Beirut. Verbal or written consent was obtained from participants before administering the interviews, facilitated through email or phone correspondence. The consent stated the objectives of the study, the purpose of the interview, and confidentiality information related to the recorded and transcribed material and that extracted content would be securely handled and anonymized where appropriate. Participants were informed of potential limitations to their participation, including time commitments, possible discomfort in discussing sensitive topics, and the non-direct influence of their input on policy decisions, while also being assured of their right to decline or withdraw at any stage without any negative consequences. The research team conducted the interviews using a uniform open-ended questionnaire tailored to each stakeholder’s expertise and role (Supplementary Appendix 1). Participant consent, audio and video recordings, or written transcripts were acquired. Interviews were conducted in English or Arabic and translated to English during the data transcription phase. All these interviews were conducted in either private offices or through the Zoom online meeting application with a minimum interviewing time of 25 min. The names of the stakeholders were not published.

### 2.5 Data analysis

Thematic analysis, as described by Braun and Clarke (2006), was employed for this study, with a focus on the sociocultural theory to identify themes related to the management of venomous bites in Lebanon (23). This approach was consistent with the sociocultural theory, focusing on how social, cultural, and economic factors influence healthcare practices and access.

After transcribing all interviews, data were systematically coded, identifying recurring themes and patterns. Initial code development focused on examining social and cultural factors related to snakebite and scorpion sting incidents, particularly inequities in medical attention, resource access, and symptom management. The transcripts were thoroughly reviewed to gain familiarity with the content, allowing for a nuanced reflection on how participant responses aligned or diverged from the initial codes.

Subsequent identification of themes within individual transcripts revealed shared experiences, such as limited access to antivenom in low-socioeconomic areas, highlighting the broader implications of inadequate healthcare access. These themes were refined to provide deeper insights into the underlying issues faced by participants in their encounters with venomous bites. Furthermore, the analysis contributed to the existing body of scholarship by introducing a social justice perspective that advocates for enhanced intervention practices and public education, particularly in historically marginalized regions.

A key focus was placed on issues of inequity, including disparities in healthcare access between urban and rural populations, the effects of economic crises on medical resources, and the impact of cultural practices on treatment-seeking behavior. This comprehensive approach facilitated an exploration of the systemic challenges and disparities inherent in managing snakebites and scorpion stings in Lebanon.

## 3 Results

In this study, a total of 17 key stakeholders were interviewed (Supplementary material). Based on the sociocultural theory and upon analyzing participants’ responses, the following several themes and sub-themes were identified and analyzed:

### 3.1 Availability and access to antivenom

The limited availability of antivenom emerged as a critical concern across various healthcare facilities, with interviewed physicians highlighting the significant challenges often necessitating referrals to other hospitals. This issue underscores inequities in healthcare access exacerbated by Lebanon’s ongoing economic crisis and financial constraints. The crisis has resulted in a 1.5-year shortage of antivenom at local health institutions, with the MoPH primarily distributing antivenom to public hospitals. Private hospitals, on the other hand, must purchase it from the ministry using their funds or source it from Lebanon’s neighboring countries like Syria. This situation creates further difficulties for hospitals unable to afford the drugs, forcing many to resort to securing antivenom through unauthorized or illicit channels. The economic crisis has not only disrupted the antivenom supply chain but also led to unconventional solutions, such as referring patients to the United Nations medical center in southern Lebanon, which uniquely maintains an antivenom stock.

*“Distribution is either through UNIFIL or black market.”*

This underscores the reliance of Lebanese hospitals on external organizations capable of importing and providing support to the local population, effectively replacing the role of the government by using stocks intended for military use.

*“There is really none, as mentioned we tend to refer patients to other hospitals whenever the anti-venom is not available. At times the adequate amount of anti-venom is not provided in order to save lives.”*

*“They (hospitals) get it from the black market or get the Indian one which is not efficient and not specific to Lebanese snakes and has higher risk of anaphylaxis.”*

*“Over the past 3–4 years we have had a national shortage of antivenom stocking, and this limited stock is mainly given to governmental hospitals… As you know we do not have in Lebanon a production of antivenom, so they import the polyvalent 2 which is a serum antivenom that works against the snakes found in Lebanon. We mainly have vipers in Lebanon. Supply of antivenom is very limited currently.”*

These findings highlight the inequity in healthcare access, where economic and logistical barriers significantly impact the management of venomous bites. ED physicians widely echoed the pattern of limited antivenom availability. The centralization of access through the MoPH might have contributed to this scarcity, with no clear policies in place to reduce or subsidize the cost of antivenom. Private importers without government affiliation have not been involved in addressing the issue, further complicating the situation.

*“The antivenom is usually available, however, in limited quantities due to its elevated cost.”*

Despite variations in bite incident rates across regions, a pronounced discrepancy in access to envenomation treatment exists, particularly affecting rural areas. Individuals in rural areas face significant challenges in accessing medical care promptly. Even with low incidence rates, limited stock availability hinders effective intervention when venomous bites occur.

Physicians pointed out that urban areas are more privileged due to the higher availability of medical care centers, while transportation remains a significant barrier for rural residents. The Lebanese economic crisis has compounded this challenge, impacting individuals’ ability to access medical services, and highlighting social equity concerns where geographic disparities impact healthcare access. This issue is further exacerbated by the higher risk of venomous bites in rural settings compared to urban ones. Finally, the centralization of high-quality, well-stocked care centers closer to the Lebanese capital makes this discrepancy more redundant.

*“Urban areas are more privileged than rural areas due to the higher availability of medical care centers, resulting in less transit time.”*

*“Transportation emerged as a significant barrier, especially for rural areas.”*

One of the interviewed physicians denoted that at times the availability of antivenom becomes so scarce that the best available assessment will be ICU observation and symptomatic treatment. This highlights the desperation of patients and physicians alike in coming up with subsidiary treatments.

*“In the case of subsidiary hospitals, most of them do not have the antivenom and at times we must refer them to other hospitals. At times we treat based on symptomatology. They request a CBC, electrolytes and PT/PTT.”*

*“They [patients] usually get treatment, stay in ICU for 24 h and then they leave.”*

### 3.2 Public education and awareness

Healthcare professionals unanimously agreed that public education on snake and scorpion bites is lacking. Physicians highlighted widespread misconceptions among the population, such as the misuse of tourniquets, burning wounds, using a cautery, or using ice packs to treat bites. Traditional practices, such as constriction bands and creating scratches at the injury site, remain prevalent in rural regions, indicating an urgent need for improved education.

*“Patients, mostly from rural areas, are familiar with the snakes in that area and are aware of the potential seriousness of a snake bite.”*

Despite these misconceptions, many victims, particularly in rural areas, are aware of the potential seriousness of snake bites. Cases typically arise either accidentally or when someone is manipulating a snake.

*“In reality, most victims are usually in a state of panic but are fully aware of the seriousness of the situation.”*

Public awareness initiatives regarding snake and scorpion bites vary significantly, with urban areas having more robust initiatives than rural ones. One physician highlighted the evolving understanding of snake species in the region, stating that many snakes initially thought to be venomous are non-venomous. This highlights the ongoing need for public education and updating guidelines tailored to the region.

The geographical distribution of bite cases was also discussed, with one health professional noting that most incidents occur in mountainous areas, affecting hospital presentation patterns. However, fewer educational initiatives are being conducted in rural areas. Moreover, none of the participants discussed preventative measures for high-risk areas, indicating a potential gap in proactive strategies to reduce venomous bites before they occur.

No cultural or religious aspects were highlighted by the stakeholders. This could be explained by the fact that primary medical care usage does not quarrel with traditional and local beliefs when it comes to the management of wildlife envenomation. Additionally, while treatment approaches may vary among physicians due to differences in training and a lack of a standardized protocol, management remains medical and supportive following the minimum standard of care, with no reliance on traditional practices.

### 3.3 Administration, diagnoses, and management of venomous bites

The lack of experience and training in managing venomous bites was evident among participants, leading to case-dependent management heavily reliant on the experience of medical providers. Physicians emphasized their reliance on individual clinical experience, managing approximately 10–12 snakebite cases annually. Concerns were expressed about the lack of knowledge and clear guidelines, resulting in inconsistent management practices. The absence of official training from the MoPH forces hospitals to develop their training programs. One physician stated that most actions during bite cases are “done on the spot” by first responders.

One physician pointed out that while expertise and training in local snakebite management are crucial, the latter is usually covered during emergency medicine training. Yet, results showed that not all physicians could differentiate between venomous and nonvenomous bites.

*“In our case, we tend to see 10–12 cases of snake bites per year, and they tend to handle it pretty well. We have had very few mortality cases since then. But is mainly based on experience.”*

*“Each hospital is forming its own training, if applicable, but there’s no official training from the MoPH regarding the snakebite.”*

Opinions varied among interviewed clinicians regarding their management capabilities and methods. One physician in a rural hospital mentioned the reliance on toxicology consultations from international hospitals, such as Emory Hospital, indicating a lack of specialized expertise in rural settings. Another physician highlighted the absence of a standardized protocol, leading to similar treatment plans for all bites. This approach not only results in the unnecessary use of medications and increases costs for both patients and hospitals but also exacerbates the shortage of antivenom reserve.

*“Training for managing snakebites is part of the emergency medicine training and its curriculum but you always need to know and have expertise in your local snakebites.”*

Indications for antivenom administration varied among participants. Some physicians emphasized symptomatology and the extent of the reaction (i.e., localized or systemic) for treatment, using corticosteroids and antihistamines for mild cases. Others followed a protocol based on clinical symptoms and vital signs, with adjustments according to severity procured from the ministry. This variation in management approaches suggests either a lack of accessibility to ministry protocols or the failure to create a standardized protocol suitable for the region’s specific obstacles.

*“We do not usually attempt to differentiate venomous from non-venomous bites, since we do not have the necessary expertise. We treat all bites under the same protocol.*”

The availability of antivenom emerged as the primary factor influencing its administration. One physician explicitly stated that the availability of antivenom renders protocols useless in its absence. Financial considerations, lack of awareness, and poor physician judgment were also mentioned as potential factors hindering the administering of antivenom when available. Another physician noted that, although ten ampoules of antivenom should be given regardless of patient weight or age, many times only two ampoules are administered for mild cases to conserve limited antivenom stocks. This practice reflects a widespread issue where many hospitals handle venomous bites incorrectly, often using antibiotics as the first-line therapy.

*“There are no hindering factors, it is mainly a physician’s judgment. Sometimes, patients present with certain perceptions that for every snake bite they should take the antivenom while that is not usually the case. In Lebanon, we have 3 venomous snakes, and they often lead to coagulation complications, but it is also possible to have a dry bite especially in the case of the vipers found in Lebanon.”*

*“There is definitely a lack of awareness between patients and even attendings, as I believe 90% of hospitals handle it wrongly. Most of them go for antibiotics but they have no role in treating and are not first line in the treatment. There is a lack of understanding for the patients, especially those who live in the city. However, some who live in the rural areas might have a better understanding and handling of the situations.”*

### 3.4 Medical reporting

Data collection and feedback mechanisms for assessing responses to venomous bites appeared limited. Although a dedicated hotline exists for reporting snake and scorpion bite injuries in Lebanon, its utilization seems inadequate. Physicians reported informing their hospital’s infectious control unit of bite cases. Still, it remains unclear whether these reports are forwarded to the MoPH and if the ministry takes any action based on these reports. This lack of government action or communication underscores a significant deficiency in public awareness regarding the symptomology associated with snakebites. Consequently, the scarcity of information available to the public about safety awareness and treatment options may deter individuals from seeking necessary medical treatment.

*“I am uncertain whether reports were being sent to the MoPH or if MoPH took any action with these reports.”*

In addition to the lack of case reporting, many physicians highlighted a deficiency in data on the quality of antivenom used to treat patients. Antivenom imported from Syria and India lacks proper monitoring and testing infrastructure, raising concerns about its efficacy and safety.

*“There is a lack of data on the quality of the antivenom imported from Syria and India, with no infrastructure present to monitor or test the imported medications.”*

This gap in quality assurance further complicates the management of venomous bites, as it adds another layer of uncertainty to an already challenging healthcare issue.

## 4 Discussion

This qualitative study examined the management of snake and scorpion bite injuries in Lebanon, focusing on how social, cultural, and economic factors influence healthcare practices.

Snakebites are a recognized global health issue, disproportionately affecting impoverished communities (24). Climate change and rising temperatures are expected to increase envenomation incidents as warmer conditions support the proliferation of these species (16). Additionally, human encroachment into natural habitats raises the risk of encounters, especially since snakes and scorpions, as ectotherms, become more active with higher temperatures (16).

The study identified and confirmed several critical challenges in managing venomous bites in Lebanon, highlighting significant disparities across regions and healthcare settings (1). A key issue is the limited availability and access to safe, quality-assured antivenom, with stock shortages being a global problem, especially in countries like Lebanon that rely on imports (25). This situation has been worsened by Lebanon’s recent socioeconomic crisis, characterized by severe inflation and reduced governmental funding, leading to shortages of essential medications like antivenom (26, 27). The COVID-19 pandemic further disrupted supply chains and redirected resources toward pandemic-related needs, compounding the difficulty in securing antivenom (28). As a result, this study confirms that Lebanon heavily depends on external aid and medical professionals often resort to illicit channels, reflecting their desperation to treat victims and willingness to stretch their resources beyond their capabilities. Many venomous bite victims face high out-of-pocket costs, leading to underuse, under-dosing, and preference for lower-cost, potentially less effective products (29, 30). These ad-hoc solutions underscore the socioeconomic disparities in access to care, especially for low-income populations, and the pressing need for timely access to safe, affordable antivenom.

Disparities in antivenom availability are also evident between urban and rural areas, as well as between public and private healthcare facilities (30–32). Effective treatment requires proximity to antivenom supplies and specialized expertise, yet these resources are often scarce in rural regions (24, 33–35). Our findings highlight that rural areas often depend on international expertise, leading to delays in treatment and potentially worse patient outcomes. Establishing stronger national, regional, and international networks among emergency physicians, clinical toxicologists, veterinarians, and herpetologists, supported by a robust data and coordination system, is crucial for improving venomous bites management (36–38). The WHO’s ‘Snakebite Information and Data Platform’ offers a useful model, but Lebanon lacks sufficient reporting and documentation, underscoring the need for a comprehensive monitoring system through the MoPH (36, 39, 40).

The study revealed a significant lack of standardized treatment protocols for venomous bites, resulting in inconsistent use of antivenom among clinicians. Discrepancies in administration were primarily attributed to insufficient training on differentiating between venomous and non-venomous bites. Treatment approaches varied widely, influenced by factors such as provider training, availability of antivenom, and the victim’s awareness and financial resources. This lack of uniformity sharply contrasts with WHO guidelines for snakebite first aid, which prohibits many documented practices, including the misuse of tourniquets (24, 41, 42). Similar knowledge gaps have been observed in other MENA countries. A recent cross-sectional study in Morocco by Alahyane et al. (43) revealed that healthcare workers demonstrated limited knowledge of first aid and treatment protocols for scorpion stings, underscoring the urgent need for standardized education and training across the region. Findings also revealed that some individuals often resort to traditional practices during envenomation incidents, despite WHO guidelines advising against such methods (24). These traditional methods, including herbal medicines and other unproven or unsafe forms of first aid, persist due to cultural beliefs, financial constraints, and geographical isolation within affected communities (24). This situation highlights a clear knowledge gap among healthcare providers, wildlife experts, and patients, extending to antivenom suppliers, as noted by one stakeholder (44). Several established guidelines provide a foundation for the Lebanese government to launch public awareness campaigns and training programs for healthcare professionals. These initiatives should be led by toxicology departments at local hospitals, in collaboration with the MoPH and WHO, to address knowledge gaps and deliver educational seminars, particularly targeting high-risk populations in rural areas.

Based on the challenges identified in managing venomous snakes and scorpion bites in Lebanon, several targeted recommendations can significantly improve outcomes. First, efforts should prioritize the equitable distribution of antivenom throughout the country, with particular emphasis on rural areas where access is limited. Second, implementing comprehensive national training programs for emergency physicians is essential to enhance their capacity to identify and treat venomous bites effectively. Collaborative initiatives involving wildlife experts should focus on raising public awareness about local snake and scorpion species, their habitats, and appropriate response measures. Moreover, partnerships with NGOs like the Lebanese Red Cross can facilitate workshops to educate both healthcare providers and community members on proper first-aid protocols. Standardizing protocols for snake and scorpion bite management across all healthcare facilities and establishing a national poison incidents registry are crucial steps toward improving overall response and treatment outcomes in Lebanon.

Several countries have implemented effective strategies to improve snakebite and scorpion sting management that could inform practices in Lebanon. In India, improved access to antivenom in rural areas significantly reduced mortality rates (45). A national program was also launched addressing snakebite prevention and treatment through public education and healthcare provider training (46). Mexico and Kenya addressed widespread scorpion and snakebite stings, respectively, through the use of locally produced region-specific antivenoms composed of highly purified antibody fragments, which are more effective and better tolerated (47, 48). In Sub-Saharan Africa, countries like Burkina Faso and Togo subsidized antivenom, while Cameroon improved surveillance through mandatory reporting (49). Moreover, a study in Morocco highlighted critical gaps in healthcare workers’ knowledge, leading to calls for enhanced training (43). These examples demonstrate that practical, context-adapted solutions are achievable and could be tailored to Lebanon’s needs.

This study presents both strengths and limitations. It offers an in-depth understanding of the multifaceted aspects of snake bites and scorpion stings from the perspectives of physicians, paramedics, and public health experts. This synthesized information is crucial for devising effective prevention and treatment strategies, which are currently lacking in Lebanon. The narratives from healthcare providers highlight the factors influencing envenomation incidents, their consequences, and the procedures within the healthcare system, as well as the limitations that hinder proper care.

However, limitations related to the data collection process must be acknowledged. First, localizing envenomation cases proved challenging due to their low incidence. Additionally, we could not interview key figures, such as the Minister of Public Health or primary care center managers, due to time constraints. Another limitation is the lack of data on wildlife envenomation cases in Lebanon, leading to the extrapolation of certain questionnaire components from data in other MENA countries. While this qualitative approach provides valuable insights, it limits generalizability. Future research should incorporate diverse perspectives and quantitative assessments to validate findings. Addressing political, economic, and healthcare infrastructure challenges is crucial, although implementation may face bureaucratic hurdles and resource constraints. Nonetheless, this study offers a qualitative framework for reducing the burden of snake and scorpion bites in Lebanon.

In conclusion, this study sheds light on the multifaceted challenges surrounding the management of venomous snakes and scorpion bites in Lebanon. Venomous bites present a real threat to local vulnerable populations. Our findings underscore the critical need for improved access to antivenom, enhanced training for healthcare providers, and comprehensive public awareness initiatives, particularly in rural areas. The disparities in healthcare access and the socioeconomic challenges exacerbated by recent crises highlight the urgency for policy reforms and strategic interventions.

The burden of snake and scorpion bites can be mitigated by implementing the proposed recommendations—including equitable distribution of antivenom, standardized training programs, collaborative public education efforts, and enhanced governmental coordination—ultimately improving outcomes for affected individuals across Lebanon. Addressing these issues requires a concerted effort from governmental bodies, healthcare professionals, community organizations, and international partners to ensure sustainable solutions and equitable healthcare access for all.

## Data Availability

The raw data supporting the conclusions of this article will be made available by the authors, without undue reservation.
