# Supplementary material for: Challenges and management of venomous bites and scorpion stings in Lebanon: a qualitative study
Source: Front Public Health. 2025 Jun 2;13:1585250. doi: 10.3389/fpubh.2025.1585250 (PMC12171459; doi:10.3389/fpubh.2025.1585250)
Supplement: Supplementary file 1 [file Data_Sheet_1.PDF]

Supplementary Material

1 Supplementary Data

1.1 Questionnaire used during the interviews in English

1.1.1 Interview Questions for ER Doctors

1.1.1.1 Available Resources

1. Is antivenom usually available in medical facilities? Is there limited access, and what about reserve stocks?

2. What experience or training do you have in treating or clinically managing venomous bites? What available resources do you have to guide treatment (society guidelines, local authorities, clinical experience…?)

3. What initiatives or programs are in place to raise public awareness about the risks associated with snake and scorpion bites?

4. What are the barriers that hinder the access of venomous bite injury victims to medical services (transportation, cost …)?

1.1.1.2 Antivenom Use

1. Are clinicians capable of distinguishing venomous from non-venomous bites to determine whether or not to use the antivenom?

2. What are the indications you use to administer the antivenom (clinical signs, laboratory findings…)?

3. What are the influencing factors in administering the antivenom (cost, prior knowledge of side effects, lack of specialized staff …)?

4. What are the reasons that hinder the physician from administering the antivenom when it is available (financial, lack of awareness, skepticism of scientific data supporting antivenom for non-life-threatening conditions…)?

1.1.1.3 Public Awareness on Bite Injuries

1. Is there a lack of education or misconceptions among patients regarding the response to bite injuries (sucking out blood at bite site, placing a tourniquet…)?

2. Do victims of bite injuries usually realize the severity of the situation?

1.1.2 Interview Questions for Healthcare Officials

1.1.2.1 Available Resources

1. Are there training sessions for healthcare providers to deal with bite injuries?
2. Is there a dedicated hotline or reporting mechanism for cases of snake and scorpion bite injuries?
3. Are specific measures implemented for the preparedness to the heightened risk of snake and scorpion bites in high-risk areas (for ex: with the increase in local temperatures)?
4. Do you collect data and feedback from medical facilities to assess the effectiveness of responses to venomous bites?

1.1.2.2 Availability of Antivenom

1. What policies and strategies are in place to ensure the availability and affordability of antivenom medications?
2. Could you explain the distribution mechanisms for antivenom across different regions of the country?
3. Is there anything done to reduce/subsidize the cost of the antivenom?

1.1.2.3 Local Population Perspective

1. Do you think time to reach medical care is causing a disproportionate burden (social equity) on individuals from rural areas?
2. Are there awareness campaigns to educate the local population on venomous animal species?
3. Is there a widely recognized and practiced protocol for immediate first aid following a bite?

1.2 Questionnaire used during the interviews in Arabic

1.2.1 أسئلة لأطباء الطوارئ

1.2.1.1 المواردة المتاحة:

1. هل يتوفّر عادةً دواء ال antivenom في المراكز الصحية؟ هل هناك محدوديّة في الحصول عليه؟ وماذا عن توافر مخزون احتياطي منه؟

2. ما هي خبرتكم أو التدريب الّذي تتلقّونه في علاج أو الاستجابة لحالات الّلدغات السامة سريريًا؟ ما هي الموارد المتاحة لديكم لتوجيه طرق العلاج والاستجابة (إرشادات مجتمعيّة، إرشادات من السلطات المحلية، خبرة سريرية ...)؟

3.ما هي المبادرات أو البرامج القائمة لزيادة الوعي العام بالمخاطر المرتبطة بلدغات الثعابين والعقارب؟

4. ما هي الحواجز التي تعيق وصول ضحايا إصابات اللدغات السامة للخدمات الطبية (النقل، التكلفة ...)؟

1.2.1.2 استخدام ال *antivenom*:

. هل لدى الأطبّاء القدرة على التمييز بين اللّدغات السامة والغير سامّة لتحديد ما إذا كان سيتم استخدام ال antivenom؟

2. ما هي المؤشرات التي يعتمدها الطبيب لإعطاء ال antivenom (مؤشرات سريريّة، نتائج مخبريّة، …)؟

3. ما هي العوامل التي تساهم في أو تعيق إعطاء ال antivenom (التكلفة، المعرفة المسبقة بالآثار الجانبية، نقص في الموظفين المتخصصين في اعطاء الدواء...)؟

4. ما هي الأسباب التي تحول دون إعطاء ال antivenom من قبل الطبيب في حال توافره (نقص الوعي، الشك في البيانات العلمية التي تدعم إعطاء ال antivenom في الظروف غير المهددة للحياة ...)؟

الوعي العام حول اللّدغات السامّة:

1. هل هناك نقص في الوعي أو مفاهيم خاطئة بين المرضى تجاه هذه اللّدغات والاستجابة لها (مصّ الدم موضع اللّدغة، وضع عاصبة ...؟)

2.هل هناك عادةً إدراك كافي لدى الضّحايا حول خطورة الحالة؟

1.2.2 أسئلة لمسؤولي الرعاية الصحية:

1.2.2.1 الموارد المتاحة

1. هل هناك دورات تدريبية لمقدمي الرعاية الصحية للتعامل مع حالات لدغات الثعابين والعقارب؟

2. هل هناك خط ساخن مخصص أو آلية إبلاغ حول حالات لدغات الثعابين والعقارب؟

3. هل يتم تنفيذ تدابير محددة للاستعداد للمخاطر المتزايدة للدغات الثعابين والعقارب في المناطق عالية الخطورة (مثلا: مع زيادة درجة الحرارة المحلية)

4. هل يتمّ جمع بيانات وتقييمات من مراكز الرعاية الصحيّة لتقييم فعالية الاستجابات للدغات السامة؟

1.2.2.2 توافر ال *antivenom*

1. ما هي السياسات والاستراتيجيات المعمول بها لضمان توافر أدوية ال antivenom والقدرة على تحمل تكلفتها؟

2. هل يمكنك شرح آلية توزيع مضادات السموم عبر المناطق مختلفة من البلاد؟

3. هل هناك أي جهود لتقليل / دعم تكلفة ال antivenom؟

1.2.2.3 منظور السكان المحليين:

1. هل تعتقد أن مدّة الوصول الى الرعاية يسبب عبئا غير إضافيًا (الإنصاف الاجتماعي) على الضحايا من المناطق الريفية؟

2. هل هناك حملات توعية لتثقيف السكان المحليين حول الأصناف الحيوانيّة السّامة؟

3. هل هناك بروتوكول معترف به وممارس على نطاق واسع للإسعافات الأولية الفورية بعد حالات اللّدغات؟

1.3 Interviewed Stakeholders

- Dr. WS, Head of internal medicine of Ain Hussain hospital and Al-Iman Hospital.
- Dr. IM, Head of emergency department at Nabih Berri Governmental University Hospital.
- Dr. NB, Emergency physician at the Al-Shahar Governmental Hospital.

- Dr. TZ, Emergency Medicine and Medical Toxicologist Attending at the American University of Beirut.
- Mrs. LZ, Red Cross paramedic.
- Dr. AG, Emergency physician at the Nini Hospital.
- Dr. HN, Emergency Physician at the Italian Lebanese Hospital.
- Dr. RF, Emergency Physician at Hiram Hospital.
- Dr. AB, Preventive Medicine Department at MoPH.
- Dr. NG, Epidemiological Surveillance Unit at MoPH.
- Dr. HZ, Toxicology laboratory at USJ.
- Mr. YN, Ministry of Environment for Explore Pesticides Use and Accidents.
- Dr. SE, Head Division of the Lebanese Red Cross in the Mount-Lebanon Area.
